# Gut microbiota and intervertebral disc degeneration: a bidirectional two-sample Mendelian randomization study

**DOI:** 10.1186/s13018-023-04081-0

**Published:** 2023-08-14

**Authors:** Ziming Geng, Jian Wang, Guangdong Chen, Jianchao Liu, Jie Lan, Zepei Zhang, Jun Miao

**Affiliations:** 1 Department of Spine Surgery, Tianjin Hospital, Tianjin University, No. 406 Jiefang South Rd, Hexi District, Tianjin, 300211 China; 2 Academy of Medical Engineering and Translational Medicine, Tianjin University, Tianjin, 300072 China

**Keywords:** Gut microbiota, Intervertebral disc degeneration, Low back pain, Causal relationship, Mendelian randomization, MiBioGen, FinnGen

## Abstract

**Background:**

Although previous studies have suggested a close association between gut microbiota (GM) and intervertebral disc degeneration (IVDD), the causal relationship between them remains unclear. Hence, we thoroughly investigate their causal relationship by means of a two-sample Mendelian randomization (MR) study, aiming to determine the impact of gut microbiota on the risk of developing intervertebral disc degeneration.

**Methods:**

Summary data from genome-wide association studies of GM (the MiBioGen) and IVDD (the FinnGen biobank) have been acquired. The inverse variance weighted (IVW) method was utilized as the primary MR analysis approach. Weighted median, MR-Egger regression, weighted mode, and simple mode were used as supplements. The Mendelian randomization pleiotropy residual sum and outlier (MR-PRESSO) and MR-Egger regression were performed to assess horizontal pleiotropy. Cochran's Q test evaluated heterogeneity. Leave-one-out sensitivity analysis was further conducted to determine the reliability of the causal relationship. A reverse MR analysis was conducted to assess potential reverse causation.

**Results:**

We identified nine gut microbial taxa that were causally associated with IVDD (*P* < 0.05). Following the Benjamini–Hochberg corrected test, the association between the phylum *Bacteroidetes* and a higher risk of IVDD remained significant (IVW FDR-corrected* P* = 0.0365). The results of the Cochrane Q test did not indicate heterogeneity (*P* > 0.05). Additionally, both the MR-Egger intercept test and the MR-PRESSO global test revealed that our results were not influenced by horizontal pleiotropy (*P* > 0.05). Furthermore, the leave-one-out analysis substantiated the reliability of the causal relationship. In the reverse analysis, no evidence was found to suggest that IVDD has an impact on the gut microbiota.

**Conclusion:**

Our results validate the potential causal impact of particular GM taxa on IVDD, thus providing fresh insights into the gut microbiota-mediated mechanism of IVDD and laying the groundwork for further research into targeted preventive measures.

**Supplementary Information:**

The online version contains supplementary material available at 10.1186/s13018-023-04081-0.

## Introduction

Low back pain (LBP), which affects a staggering 70–85% of individuals at some point in their lives, presents a significant global public health challenge, resulting in a considerable financial burden on healthcare and social systems [1–3]. In general, LBP refers to discomfort, tension or inflexibility that is felt in the region of the body situated beneath the ribcage and above the inferior gluteal folds, often accompanied by leg pain (sciatica) and other neurological issues affecting the lower extremities [1, 4]. While various factors can play a role in the development of LBP, intervertebral disc degeneration (IVDD) stands out as one of the primary causes [4, 5]. IVDD serves as the pathological foundation for various spinal degenerative disorders and is a prevalent orthopedic condition that contributes to a reduced quality of life [6]. The intervertebral disc (IVD) consists of the nucleus pulposus (NP), annulus fibrosus, and the cartilage endplate, which are primarily composed of collagen and proteoglycan, imparting crucial properties to the disc. The NP is a critical component of the IVD, primarily made up of NP cells and the extracellular matrix (ECM). Intervertebral disc degeneration (IVDD) is a prevalent degenerative condition that is distinguished by the gradual reduction of proteoglycans and water content within NP [7]. As the disease progresses, the discs between the vertebrae may break down, rendering them more susceptible to herniation, which can cause compression of the spinal nerves and nerve roots. The irritation of nerves in the lower back as a result of IVDD is known as lumbar radiculopathy. If this occurs in the nerve roots of L4-S2, it commonly results in a distinct type of pain known as sciatica [8, 9].

The gut microbiota (GM) refers to the distinct microbial populations that inhabit the intestinal tract and coexist in a mutually beneficial relationship with the host organism, including bacteria, protozoa, fungi, archaea, and viruses [10]. It has the potential to influence multiple physiological processes, including metabolism, inflammation, and immune responses [11–14]. The identification of gut microbiota taxonomic characteristics and their potential role is mainly based on the utilization of 16S rRNA and metagenomic sequencing methods, which are commonly employed techniques [15]. In a recent study by Rajasekaran et al. [16], a total of 24 lumbar intervertebral discs (IVDs) were analyzed, revealing that the microbial makeup present in healthy IVDs contrasted with that of degenerated and herniated IVDs. Changes in the composition of the microbiome and the way hosts respond to microbiota, which can cause abnormal bone growth and resorption [17, 18], gave rise to the idea of the gut-bone marrow axis [19, 20] and the gut-bone axis [18]. Subsequent to the study conducted by Rajasekaran et al., a comparable gut-disc axis concept has emerged that could have significant implications in intervertebral disc degeneration and low back pain [16, 21]. As a result, the regulation of gut microbiota could potentially impact the diversity and quantity of microbiota within the intervertebral disc, ultimately helping to regulate intervertebral disc degeneration.

However, additional investigation is required to further explore the distinct role of various gut microbiota taxa in the development of intervertebral disc degeneration. Akin to randomized controlled trials (RCT), the Mendelian randomization (MR) study is a recent research approach that investigates the causal relationship between exposure and outcome [22]. Mendelian randomization is a genetic epidemiology technique that uses single nucleotide polymorphisms (SNPs) that are known to affect modifiable exposures as instrumental variables (IVs) to deduce the causal effect of an exposure on an outcome. This approach is advantageous because it can eliminate confounding bias and can help to distinguish between the causal pathways of phenotypically grouped risk variables that are difficult to randomize or that are prone to measurement error [23]. In this study, we utilized GWAS summary statistics of GM and IVDD to perform MR analysis, with the aim of identifying GM taxa that may have a significant impact. This approach can help to confirm existing evidence and offer fresh perspectives on the management and prevention of intervertebral disc degeneration.

## Materials and methods

### Study design

**Fig. 1:**
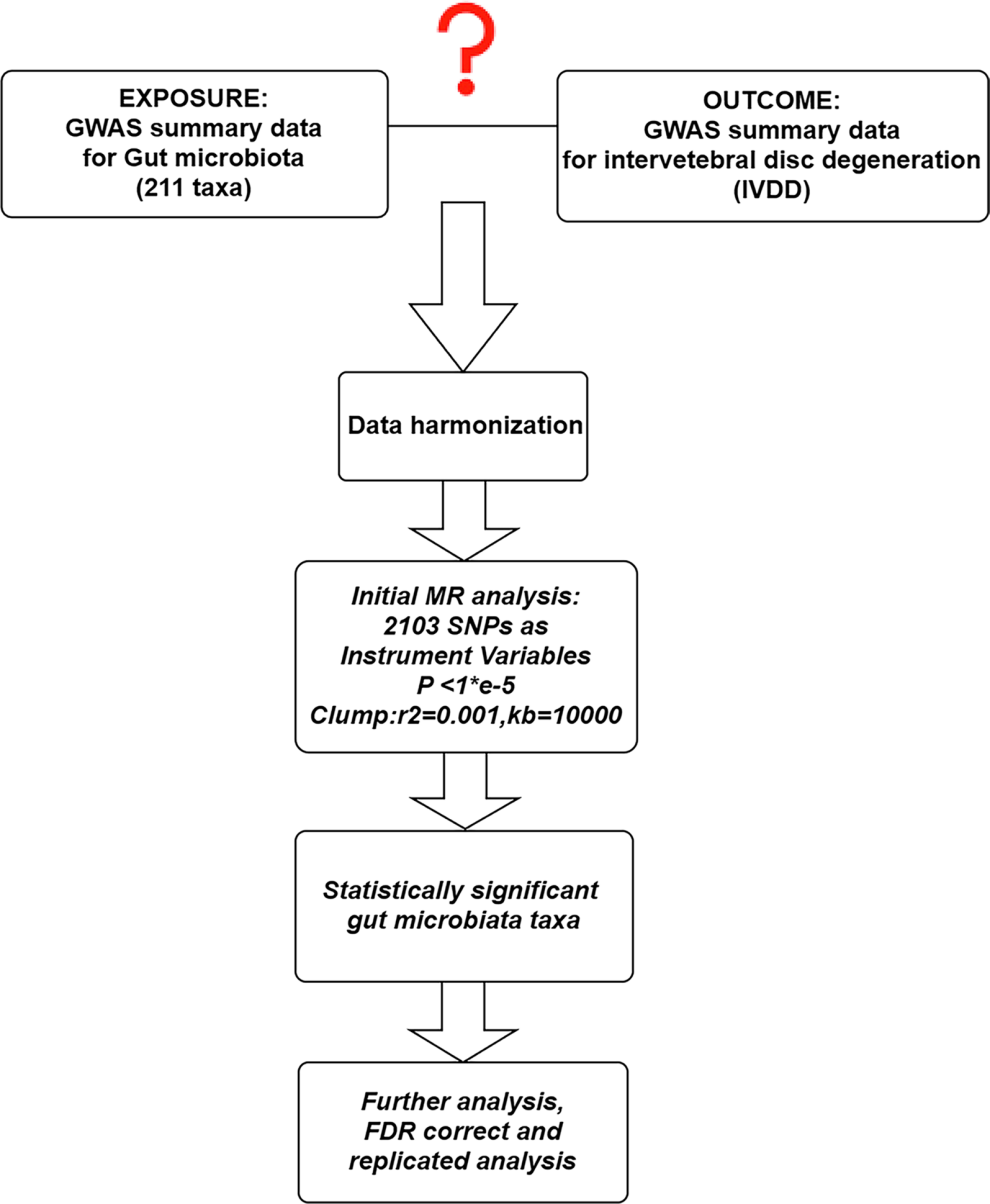
Overall flow chart of this study

The overall flowchart of this study is shown in Fig. 1. MR studies require three assumptions to be met: (1) a strong correlation between the instrumental variable (IV) and the exposure, (2) IVs are unrelated to confounding factors, and (3) IVs are only related to the outcome through the exposure [24, 25]. Specifically, we determined the gut microbiota taxa that had a causal effect on intervertebral disc degeneration (IVDD) through bidirectional two-sample Mendelian randomization. Our results were reported according to the STROBE-MR guidelines [26]. We utilized GWAS data that had previously been obtained with informed consent and ethical approval for public release.

### Data sources for exposure and outcome

Based on twin, family, and population-based studies, it is evident that genetic factors also play a role in determining the composition of the gut microbiota, and some bacterial taxa exhibit heritability [27, 28]. The MiBioGen consortium conducted a study analyzing the genotypes of hosts and the sequencing profiles of 16S fecal microbiomes rRNA gene of 18,340 participants, as reported by Kurilshikov et al. [11]. The 18,340 participants in this study were sourced from 24 cohorts spanning the United States, Canada, Israel, South Korea, Germany, Denmark, the Netherlands, Belgium, Sweden, Finland, and the United Kingdom. Microbiome trait loci (mbTL) were identified using standardized methods to pinpoint genetic loci that influence the relative abundance (mbQTLs) or presence (mbBTLs) of specific microbial taxa. Finally, Gene Set Enrichment Analysis (GSEA) and Phenome-wide association studies (PheWAS) were conducted to provide biological interpretations of the results from the genome-wide association study (GWAS). The GWAS study examined 211 GM taxa ranging from genus to phylum level and discovered genetic variants associated with 9 phyla, 16 classes, 20 orders, 35 families, and 131 genera.

We obtained summary statistics of GWAS for IVDD from the FinnGen Consortium R8 release, which included 29,508 cases and 227,388 controls [29]. The FinnGen project was initiated in the autumn of 2017 in Finland, aiming to integrate genomic information with digital healthcare data and involving collaboration from universities, hospitals, THL (National Institute for Health and Welfare), blood service centers, biobanks, FINBB (Finnish Biobanks), international pharmaceutical companies, and hundreds of thousands of Finnish participants. The primary goal of this project is to collect and analyze genomic and health data from 500,000 participants in the Finnish Biobank to enhance human health and discover novel approaches for treating various diseases. The data collected includes individual clinical medical information, genomic data, and environmental/lifestyle factors. To establish an extensive and comprehensive research resource, FinnGen employs various data collection methods, including medical information from Finland’s national healthcare archives and genomic data obtained through large-scale gene sequencing and genotyping techniques. Additionally, the project may also gather disease-related survey questionnaires and biological specimens to obtain more comprehensive data information. The diagnosis of IVDD was based on ICD-10 M51, ICD-9 722, and ICD-8 275, excluded ICD-9 7220|7224|7227|7228A, ICD-8 7250. Table 1 presents detailed information on the exposure and outcome analyzed in this MR study. The details of the exposure and outcome are shown in Table 1.

**Table 1:** Details of the exposure and outcome

Trait	Consortium	Samples	Case	Control
*Exposure*				
211 GM taxa	MiBioGen	18,340	/	/
*Outcome*				
Intervertebral disc degeneration	FinnGen (R8)	256,896	29,508	227,388

### Identification of IVs

SNPs closely associated with each GM taxon were used as instrumental variables (IVs) in this MR study. Due to the limited number of IVs obtained at a strict threshold (*P* < 5 × 10^−8^), a more comprehensive threshold (*P* < 1 × 10^−5^) was utilized to obtain a relatively higher number of IVs, thus resulting in more robust results [30]. In addition, to ensure the independence of each IV, SNPs within a 10,000 kb window size with a threshold of *r*^2^ < 0.001 were pruned to mitigate linkage disequilibrium (LD). Subsequently, we eliminated palindromic SNPs and SNPs that did not appear in the outcome from the IVs. Ultimately, we computed the F statistic for the IVs to evaluate the degree of bias due to weak instruments.The calculation formula is as follows: *F* = $\frac{N - K - 1}{K} \times \frac{{R^{2} }}{{1 - { }R^{2} }}$; *R*^2^ = 2 × EAF × (1 − EAF) × BETA^2^ [31]. *R*^2^ represents the proportion of variance in the exposure that is explained by genetic variants. N = sample size; K = the number of IV. If the F statistic > 10, weak IVs were deemed not to have caused bias [32].

### Statistical methods

For each GM taxon, the inverse variance weighted (IVW) method was used as the primary analysis method to determine causal associations (*P* < 0.05), with four additional methods (MR-Egger, weighted median, simple mode, and weighted mode) employed as supplementary measures [33, 34]. This study conducted sensitivity analyses in order to eliminate potential bias and examine the robustness of the IVW results. The Cochran Q test was utilized to assess heterogeneity among SNPs, and a *P* value greater than 0.05 indicates a lower likelihood of heterogeneity among the SNPs, in which case the IVW fixed-effect model was employed for analysis. Conversely, if the *P* value was less than or equal to 0.05, the IVW random-effects model was used [35].

In IVW regression, the intercept term is not considered and the reciprocal of the outcome variance (se^2^) is used as weights for fitting [33]. The weighted median method is defined as the median of the weighted empirical density function of the ratio estimates, and causal relationships can be consistently estimated if at least 50% of the information in the analysis comes from valid instruments [33]. In MR-Egger regression, the intercept term is considered, and the reciprocal of the outcome variance (se^2^) is also used as weights for fitting, with the resulting intercept used to assess horizontal pleiotropy [36]. MR-PRESSO global test was also utilized to achieve the same objective, which eliminated the influence of pleiotropy by removing outliers [37]. In addition, funnel plots and forest plots were constructed to visualize and ensure the reliability of the results. Finally, we converted the effect estimates to odds ratios (ORs) and their corresponding 95% confidence intervals (CIs) to more intuitively display the causal associations between each GATA taxon and outcomes.

A significance level of *P* < 0.05 indicated the presence of a causal relationship between the exposure and outcome.To account for multiple testing (multiple exposures), the significance of the MR effect estimates was controlled using a Benjamini–Hochberg false discovery rate (FDR) of < 5% at a specific level. Additionally, we performed reverse causal analysis to examine the reverse causality relationship. To meet the core assumptions of MR, the selected SNPs were further filtered in the Phenoscanner database to ensure that the included instrumental variables were not correlated with known confounding factors [38], including dried fruit intake [39], diabetes [40], insomnia [41], plasma omega-3 levels [42], obesity [43], smoking. The replicated MR analyses were performed after excluding instrumental variables associated with confounding factors mentioned earlier. All statistical analyses were performed using the “TwoSampleMR” package and the “MRPRESSO” package in R language (version 4.3.0).

## Results

### Selection of instrumental variables

After excluding three unknown families and twelve unknown genera from a pool of 211 gut microbiota (GM) taxa, a total of 196 GM taxa were included as exposure. There are 2103 SNPs were selected as instrumental variables, which can be categorized according to five levels: 185 SNPs in 16 classes, 353 SNPs in 32 families, 1232 SNPs in 119 genera, 227 SNPs in 20 orders, and 106 SNPs in 9 phyla. All instrumental variables showed F-values greater than 10, indicating their resilience to weak instrumental variables. Detailed information regarding the instrumental variables can be found in the Additional file 1: Table S1.

### Causal effects of gut microbiota on intervertebral disc degeneration

Causal Associations from 9 GM taxa (2 Families, 6 Genera, and 1 Phylum) to IVDD were identified through Mendelian randomization analysis evaluating the causal link between microbiota taxa at five Levels and IVDD (Additional file 1: Table S2).

**Fig. 2:**
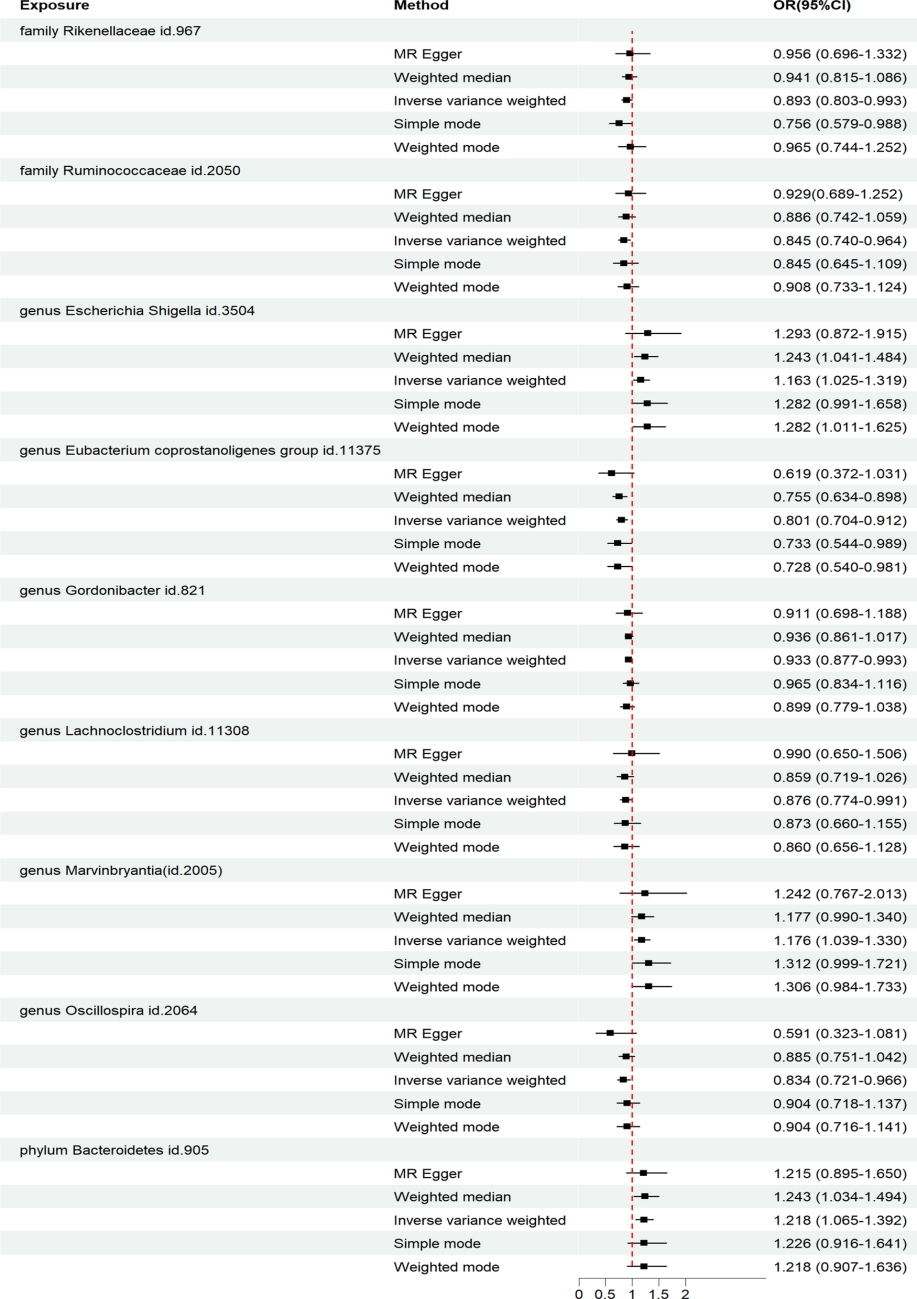
Diverse Mendelian randomization (MR) results for 9 GM taxa causally associated with IVDD

As shown in Fig. 2, the IVW analysis revealed that the *genus Escherichia Shigella* (OR: 1.163, 95% CI 1.025–1.319, *P* = 0.019), *genus Marvinbryantia* (OR: 1.176, 95% CI 1.039–1.330, *P* = 0.010), and *phylum Bacteroidetes* (OR: 1.218, 95% CI 1.065–1.392, *P* = 0.004) were associated with an increased risk of IVDD. On the other hand, the *family Rikenellaceae* (OR: 0.893, 95% CI 0.803–0.993, *P* = 0.037), *family Ruminococcaceae* (OR: 0.845, 95% CI 0.740–0.964, *P* = 0.005), *genus Eubacterium coprostanoligenes group* (OR: 0.801, 95% CI 0.704–0.912, *P* < 0.001), *genus Gordonibacter* (OR: 0.933, 95% CI 0.877–0.993, *P* = 0.028), *genus Lachnoclostridium* (OR: 0.876, 95% CI 0.774–0.991, *P* = 0.035), and *genus Oscillospira* (OR: 0.834, 95% CI 0.721–0.966,* P* = 0.016) were associated with a reduced risk of IVDD.

In addition, we performed four supplementary analysis methods to complement the IVW analysis, and the results were consistent with the direction of the IVW method (Fig. 2). This indicates the robustness of the IVW results.

### Benjamini–Hochberg corrected test, sensitivity analysis and reverse analysis

After conducting the Benjamini–Hochberg corrected test, it was found that the phylum *Bacteroidetes* remained associated with a higher risk of IVDD (IVW FDR-corrected *P* = 0.037) (Additional file 1: Table S2). No heterogeneity was observed according to the Cochrane Q and MR-Egger tests, and the analysis results of MR-Egger and MRPRESSO global tests indicated the absence of horizontal pleiotropy (Table 2).

**Table 2:** Results of heterogeneity and horizontal pleiotropy analysis for IVs of 9 GM taxa associated with IVDD

Exposure	Heterogeneity test		MR-Egger intercept test			MR-PRESSO global test	
	MR-IVW	MR-Egger	Egger_intercept	SE	*P*	RSS obs	*P*
*Family*							
Family Rikenellaceae id.967	Q = 14.471	Q = 14.289	− 0.005	0.012	0.676	16.364	0.565
	*P* = 0.564	*P* = 0.504					
Family Ruminococcaceae id.2050	Q = 6.676	Q = 6.189	− 0.010	0.014	0.508	8.5467	0.623
	*P* = 0.572	*P* = 0.518					
*Genus*							
Genus Escherichia Shigella id.3504	Q = 7.955	Q = 7.645	− 0.008	0.015	0.593	9.708	0.542
	*P* = 0.539	*P* = 0.469					
Genus Eubacterium coprostanoligenes group id.11375	Q = 7.735	Q = 6.683	0.016	0.016	0.327	9.066	0.761
	*P* = 0.806	*P* = 0.824					
Genus Gordonibacter id.821	Q = 6.773	Q = 6.740	0.004	0.020	0.858	8.198	0.775
	*P* = 0.817	*P* = 0.750					
Genus Lachnoclostridium id.11308	Q = 11.166	Q = 10.808	− 0.009	0.014	0.562	12.955	0.517
	*P* = 0.515	*P* = 0.460					
Genus Marvinbryantia id.2005	Q = 6.462	Q = 6.409	− 0.005	0.021	0.824	8.243	0.716
	*P* = 0.693	*P* = 0.602					
Genus *Oscillospira* id.2064	Q = 10.485	Q = 8.580	0.034	0.030	0.292	13.456	0.155
	*P* = 0.163	*P* = 0.199					
*Phylum*							
Phylum *Bacteroidetes* id.905	Q = 8.994	Q = 8.994	0.000	0.011	0.991	10.871	0.564
	*P* = 0.533	*P* = 0.438					

The leave-one-out sensitivity analysis showed that the IVW analysis results remained similar when any SNP was removed as instrumental variable (Additional file 2: Fig. S1). In the reverse MR analysis, we found no significant causal effect of IVDD on gut microbiota (Additional file 1: Table S3).

### Further analysis for removing potential confounding factors

Among the instrumental variables (IVs) associated with the 9 microbial taxa causally linked to intervertebral disc degeneration (IVDD), rs55793120, rs72829893, rs12925026, and rs62532512 were found to be associated with obesity. Furthermore, rs62532512 was also found to be associated with smoking. After excluding these IVs and conducting the analysis again, it was observed that three GM taxa, including *family Rikenellaceae* (*P* = 0.0687), *genus Lachnoclostridium* (*P* = 0.0751), and *genus Oscillospira* (*P* = 0.0582), were no longer causally associated with IVDD. However, the causal associations between the remaining GM taxa and IVDD remained robust (Table 3).

**Table 3:** Replicated MR analysis by IVW method after removing confounders-related IVs

Exposure	Outcome	*P* value	OR(95%CI)
*Family*	IVDD		
Family Rikenellaceae id.967		0.069	0.903(0.809–1.008)
Family Ruminococcaceae id.2050		0.005	0.813(0.705–0.939)
*Genus*			
Genus Escherichia Shigella id.3504		0.019	1.163(1.025–1.319)
Genus Eubacterium coprostanoligenes group id.11375		0.001	0.801(0.704–0.912)
Genus Gordonibacter id.821		0.028	0.933(0.877–0.993)
Genus Lachnoclostridium id.11308		0.075	0.885(0.774–1.012)
Genus Marvinbryantia id.2005		0.010	1.176(1.039–1.330)
Genus *Oscillospira* id.2064		0.058	0.885(0.779–1.004)
*Phylum*			
Phylum *Bacteroidetes* id.905		0.004	1.218(1.065–1.392)

## Discussion

To the best of our knowledge, this is the first Mendelian randomization study investigating the causal relationship between gut microbiota (GM) and intervertebral disc degeneration (IVDD). In this study, we utilized GM data derived from a GWAS meta-analysis conducted by the MiBioGen consortium and IVDD data from the R8 release of the FinnGen consortium. The causal effects of gut microbiota taxa (from phylum to genus level) on intervertebral disc degeneration were investigated, and nine gut microbiota taxa were identified to have a causal association with IVDD. Additionally, we performed reverse analysis to demonstrate the unidirectionality of the causal relationships and found no evidence of IVDD affecting the gut microbiota.

There is a growing interest in understanding the pathogenic impact of the microbiome in numerous human diseases. Dysbiosis of the gut microbiota can potentially impair the normal functioning of the gut microbial community in maintaining host health. It may also lead to the selective enrichment of certain microbial members, including pathogenic bacteria, resulting in a dysregulated production of microbial-derived products or metabolites that can be harmful to the host. This dysregulation can contribute to the development of various diseases in local, systemic, or distant organs [44]. Similarly, it has been established that alterations in the composition of the gastrointestinal, skin, and oral microbiota are associated with various musculoskeletal diseases, such as rheumatoid arthritis [45–47], osteoarthritis [48, 49], ankylosing spondylitis [47], spondyloarthritis [50]. As one of the most common complaints in orthopedic clinics, low back pain is a prevalent public health issue worldwide, causing severe lifelong disability and imposing significant economic burdens on both patients and society [51]. Although the etiology of LBP is diverse [52–54], IVDD is widely acknowledged as a prominent factor among the various causes [4, 55], which is responsible for approximately 40% of symptomatic LBP [56]. In the past, the intervertebral discs in healthy individuals were believed to be sterile. However, Rajasekaran et al. [16] conducted a comprehensive metagenomic analysis of lumbar intervertebral discs, revealing the presence of a human intervertebral disc microbiome, and documenting the existence of “dysbiosis”. Furthermore, it was discovered that the gut and intervertebral disc microbiomes share a total of 58 bacterial species. In addition, the intervertebral disc (IVD) is a complex fibrocartilaginous joint and is often referred to as the largest avascular structure in the human body. Blood vessels in the IVD are only present in the longitudinal ligaments and the outer layers of the annulus fibrosis. However, the formation of new blood vessels can occur when the following conditions arise: intervertebral disc herniation into the extradural space, physical injury and fractures, or local inflammation on the intervertebral disc and vertebral endplates [57]. In the mouse model of IVDD, the abundance of Muribaculaceae and Lactobacillus increased, while the abundance of Clostridia_UCG-014 decreased. Furthermore, fecal microbiota transplantation further increased the abundance of Lactobacillus and reduced the abundance of Clostridia_UCG-014 [58]. Su et al. [59] conducted a Mendelian randomization study on the potential causal effects of specific gut microbiota and gut microbiota metabolites on low back pain (LBP). Similar to our findings, they observed that the abundance of the genus Marvinbryantia is a potential risk factor for LBP, while the abundance of the family Rikenellaceae and family Ruminococcaceae is a potential protective factor for LBP. These studies provide evidence for the association between gut microbiota and intervertebral disc degeneration (IVDD).

The pathogenic mechanisms underlying the role of gut microbiota in IVDD have been widely discussed in previous studies, involving various aspects such as inflammatory response, gut barrier function, and nutrient metabolism. These mechanisms intersect and collectively contribute to the overall impact of gut microbiota in IVDD. The *Escherichia Shigella* is a group of bacteria capable of producing lipopolysaccharide (LPS). LPS is a glycolipid component found in the outer membrane of gram-negative bacteria [60]. LPS can activate the TLR4/MyD88/NF-κB signaling pathway, leading to the release of pro-inflammatory mediators such as IL-6, IL-1β, and TNF-α. This cascade of events triggers a series of inflammatory processes, ultimately resulting in chronic low-grade inflammation [61]. A significant decrease in the abundance of a short chain fatty acids-producer, *Marvinbryantia* spp, was observed in the low muscle mass elders [62]. Sarcopenia is postulated to be an influential factor in chronic low back pain [63, 64]. The GM taxon may potentially impact intervertebral disc degeneration and trigger lower back pain by regulating nutrient absorption in the intestinal epithelium, thereby affecting muscle mass. *Ruminococcus*, originally classified as a member of the core phylum *Firmicutes* in the human gut microbiota, accounting for approximately 30% of the total gut microbial population [65], has been recently reclassified as *Blautia* [66]. *Blautia* is a genus of anaerobic bacteria that exhibits probiotic characteristics. It has the potential to reduce the production of local inflammatory factors and alleviate inflammatory responses in the gut. In normal intervertebral disc (IVD) samples, *Firmicutes* are abundantly present, and their abundance is associated with intestinal barrier function and antimicrobial protection [16]. Both *Firmicutes* and *Bacteroidetes* are capable of fermenting dietary fiber to produce short-chain fatty acids (SCFAs), which then act on G-protein-coupled receptors, such as GPR43, GPR41, and GPR109a,to regulate immune responses [67]. This leads to an increase in regulatory T cells (Tregs) and dendritic cell precursors, improvement in epithelial barrier function, and an upregulation of anti-inflammatory cytokines such as IL-10 [68]. The ratio of *Firmicutes* and *Bacteroidetes* has been implicated that some diseases like T2DM, hyperlipidemia [69], obesity [70], and non-alcoholic fatty liver disease (NAFLD) [71] are associated with elevated *Firmicutes* to *Bacteroidetes* ratio (F/B) in the gut microbiota. Our research, along with previous studies, demonstrates a strong association between *Firmicutes*, *Bacteroidetes* and intervertebral disc degeneration (IVDD). Thus, the F/B ratio could potentially be utilized as an evaluative indicator for assessing the association with IVDD. In mouse models of lumbar disc herniation, the gut was found to have a higher abundance of Ruminococcaceae [72], which can alleviate chronic inflammation through the production of butyrate [73]. However, it contradicts our study results to some extent, which suggest that this bacterium acts as a protective factor against IVDD. The reason for this discrepancy may be attributed to the complex interactions that exist among gut microbiota. Further prospective randomized controlled trials may be necessary to validate it. The *Gordonibacter*, as a probiotic, has been shown to alleviate inflammatory reactions and prevent the occurrence of various diseases [74]. It is known to produce anti-inflammatory metabolites, including urolithins. Urolithin A, in particular, has been found to improve mitochondrial health, reduce cartilage degeneration, and alleviate pain in osteoarthritis [75]. Studies have indicated that Urolithin A affects intervertebral discs through mechanisms that include inhibiting the breakdown metabolism of nucleus pulposus cells by TNFα in the body [76].Additionally, it increases mitochondrial autophagy and reduces cell apoptosis in the nucleus pulposus cells [77]. These findings suggest that *Gordonibacter* may have potential characteristics in regulating intervertebral disc health through the modulation of anti-inflammatory metabolites. Supplementation of vitamin D has been shown to impact the gut microbiota [78]. Importantly, vitamin D is closely associated with the gut barrier and can improve barrier function by inducing the expression of E-cadherin and enhancing epithelial cell connections within the intestine [79]. Deficiency in vitamin D can lead to disruption of these connections, resulting in intestinal permeability and facilitating the passage of bacterial components and metabolites. In patients with vitamin D deficiency and osteoarthritis, there is an increase in the abundance of *Gordonibacter* [80]. This particular microbial group may alter intestinal permeability, thereby creating conditions for the gut microbiota to influence the microecology of intervertebral discs. Yang et al. discovered that *Oscillospira* sp. constitutes a significant proportion of the gut microbiota. This suggests that *Oscillospira* sp. may play a crucial role in maintaining microbial balance and human health [81]. Importantly, its abundance has been found to be negatively correlated with a range of inflammatory diseases [82].

In the subsequent analysis after removing the instrumental variables associated with confounding factors, the analysis results for *Eubacterium coprostanoligenes group*, *Rikenellaceae*, and *Lachnoclostridium* no longer showed statistical significance.Limited research has been conducted regarding the association between *Eubacterium coprostanoligenes group*,*Rikenellaceae*, *Lachnoclostridium*, and IVDD. Therefore, whether there is a causal relationship between these three microbial taxa and IVDD remains to be debated.In conclusion,our research findings suggest that gut microbiota a dual role, both inducing and protective, in the occurrence and progression of IVDD.The specific mechanisms underlying these effects and the intricate interplay between gut microbial communities require further elucidation.

In previous studies, the etiology of intervertebral disc degeneration (IVDD) has mainly focused on various pathological factors, such as aging, inflammation, oxidative stress, mitochondrial dysfunction, and abnormal mechanical load [83–86]. However, there is limited research on the role of gut microbiota in IVDD. To the best of our knowledge, this study represents the first investigation of this topic using a two-sample bidirectional Mendelian randomization (MR) approach. MR utilizes genetic variations as instrumental variables to analyze the causal relationship between exposure and disease outcomes. By leveraging the random allocation of genetic variations, which occurs before the onset of disease and is independent of environmental factors, MR overcomes the inherent limitations of traditional observational studies, such as confounding factors and reverse causality. In this study, we employed available exposure and outcome data from genome-wide association studies (GWAS) to obtain precise analytical results. This approach provides an efficient means of harnessing reliable genetic information without the need for additional experimental costs. Furthermore, we meticulously examined the instrumental variables associated with the positive microbial results using the Phenoscanner V2 website to mitigate the influence of confounding factors and applied false discovery rate (FDR) correction to control the occurrence of false positives, thereby enhancing the reliability and reproducibility of the analysis results. Our research results provide the initial evidence of a causal association between the phylum *Bacteroidetes* and IVDD, thereby further confirming the concept of the gut-disc axis.

Our study provides insights into the relationship between specific GM taxa and intervertebral disc degeneration from a genetic perspective. By exploring potential genetic factors and the role of GM, our research contributes significantly to understanding the complex interplay between host genetics, GM, and intervertebral disc degeneration. Based on our findings, clinicians can innovate in the diagnosis and treatment of intervertebral disc degeneration in future research. Firstly, our study identifies potential therapeutic targets: by pinpointing genes and microbial taxa associated with intervertebral disc degeneration, we provide potential targets for drug development and interventions. This could lead to new treatment approaches and preventive measures aimed at modulating the genetic regulation of the microbiome or specific GM taxa to alleviate intervertebral disc degeneration. Secondly, personalized medicine approaches: the genetic insights from our research may facilitate the application of personalized medicine in managing intervertebral disc degeneration. Further investigations can explore the significance of individual-specific microbial and genetic characteristics in assessing the risk of developing intervertebral disc degeneration, allowing tailored treatment strategies. Thirdly, interdisciplinary research: our findings may inspire further collaboration between microbiologists, geneticists, and orthopedic experts. This interdisciplinary approach can provide new insights into the role of intervertebral disc degeneration and different GM taxa, bridging knowledge gaps between fields. Lastly, influencing lifestyle and environmental factors: the associations between specific microbial taxa and intervertebral disc degeneration can stimulate research on the impact of lifestyle factors (such as diet, exercise, and stress) on the GM and their potential effects on intervertebral disc health. Ultimately, evidence-based recommendations can be provided for patients with intervertebral disc degeneration. In conclusion, the genetic perspective highlighted in our study on the GM's role in intervertebral disc degeneration can guide new diagnostic and treatment strategies, support personalized medicine approaches, and foster interdisciplinary research collaboration. The results also underscore the importance of considering environmental and lifestyle factors that may influence the GM in the management of intervertebral disc degeneration.

Nevertheless, the study has several limitations. First, due to the use of a stringent genome-wide significance threshold (*P* < 5 × 10^−8^), the number of SNPs available for analysis was limited. Only SNPs meeting the suggestive significance threshold (*P* < 1 × 10^−5^) were included, which may reduce the reliability and accuracy of the results. Furthermore, the sample size and strain-level information of gut microbiota GWAS data are still in the early stages. This resulted in the exclusion of certain microbial species from the study, and the limited number of instrumental variables used may lead to a decrease in statistical power and increase the potential impact of weak instrumental variables on reverse Mendelian randomization, making it difficult to fully exclude reverse causality. It is essential to acknowledge that the study's data were exclusively obtained from European populations, without considering factors like gender and ethnicity, limiting the generalizability of the findings to other populations. Additionally, it is important to recognize that the study focused solely on bacteria, disregarding the considerable diversity of eukaryotic viruses and prokaryotic phages present in the human microbiota [87]. Further investigations are warranted to explore their potential involvement in IVDD. Finally, the association between human microbiota and the host in both healthy and disease states is a complex interplay rather than a simple one-way “causal relationship” [44]. Therefore, future studies should consider the intricate coordination and crosstalk between the host and gut microbiota to gain a better understanding of the relationship between gut microbiota and disease.

## Conclusion

Using publicly available GWAS data, we conducted a bidirectional two-sample Mendelian randomization analysis on the causal association between 211 gut microbiota taxa and intervertebral disc degeneration (IVDD). Our analysis resulted in the identification of eight nominal causal associations and one strong correlation, further providing a theoretical foundation for the concept of the gut-disc axis. This study was based on a GWAS meta-analysis dataset generated from 16S rRNA sequencing, thus highlighting the need for analyses based on more advanced large-scale studies using metagenomics sequencing. Nevertheless, our research also offers valuable biomarkers for understanding the progression, diagnosis, and potential therapeutic approaches for IVDD.

### Supplementary Information

**Additional file 1.** **Table S1.** Instrument Variables for all gut microbiota taxa. **Table S2.** Casual effects of MR analysis between gut microbiota and intervertebral disc degeneration. **Table S3.** Reverse MR analysis for the causal effect of intervertebral disc degeneration on gut microbiota.

**Additional file 2.** **Fig. S1.** Leave-one-out analysis for 9 GM taxa on IVDD.

## Data Availability

Please contact the corresponding author for data requests.

## Abbreviations

IVDD: Intervertebral disc degeneration
IVD: Intervertebral disc
NP: Nucleus pulposus
ECM: Extracellular matrix
GM: Gut microbiota
LBP: Low back pain
MR: Mendelian randomization
GWAS: Genome-wide association studies
IVW: Inverse variance weighted
WME: Weighted median
MR-PRESSO: Mendelian randomization pleiotropy residual sum and outlier
SNP: Single nucleotide polymorphism
IV: Instrumental variable
FDR: False discovery rate
