# Supplementary figures and images for: Gut microbiota and intervertebral disc degeneration: a bidirectional two-sample Mendelian randomization study
Source: J Orthop Surg Res. 2023 Aug 14;18:601. doi: 10.1186/s13018-023-04081-0 (PMC10424333; doi:10.1186/s13018-023-04081-0)

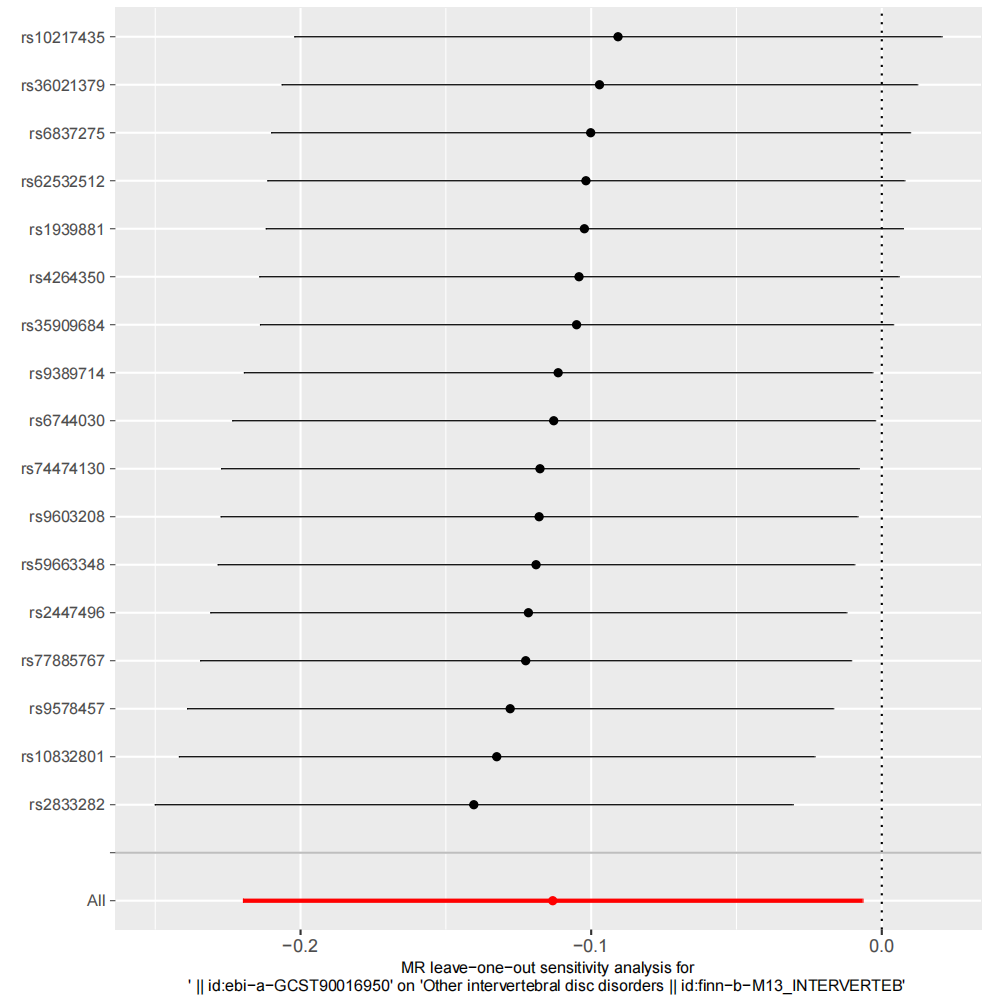

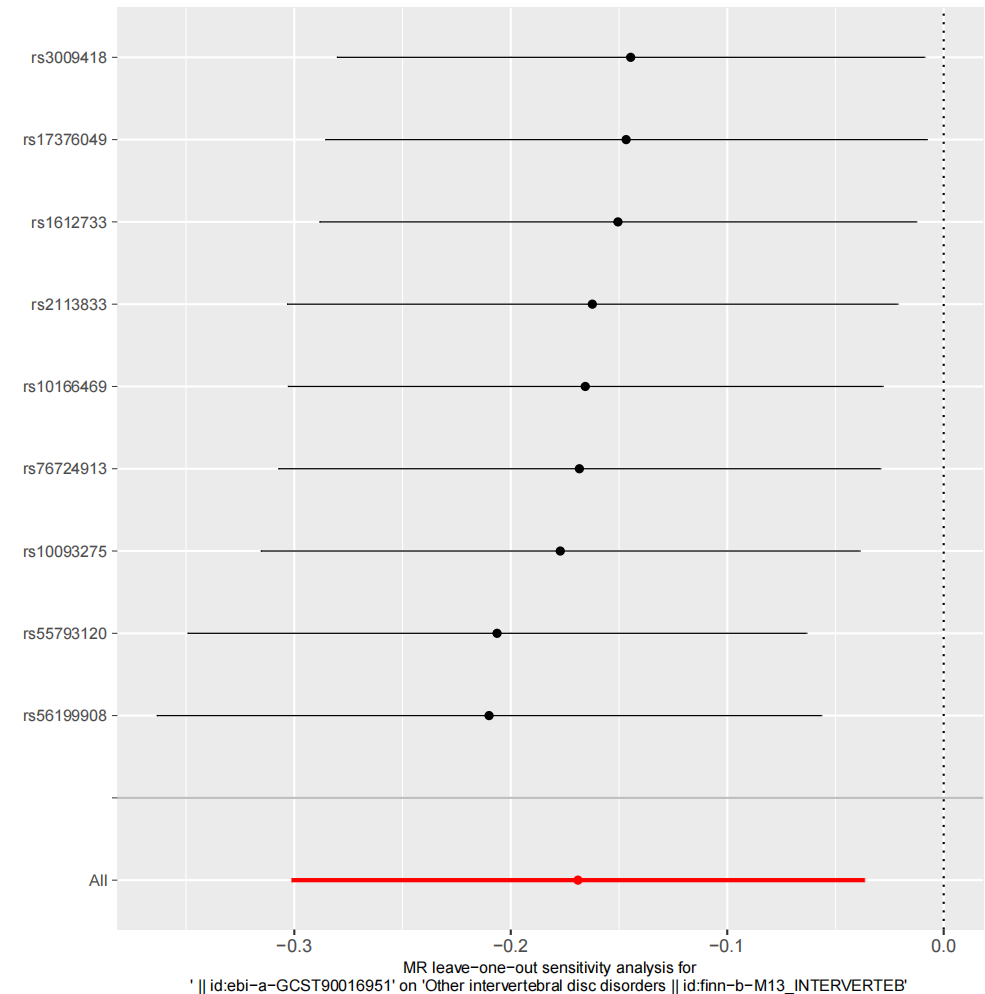

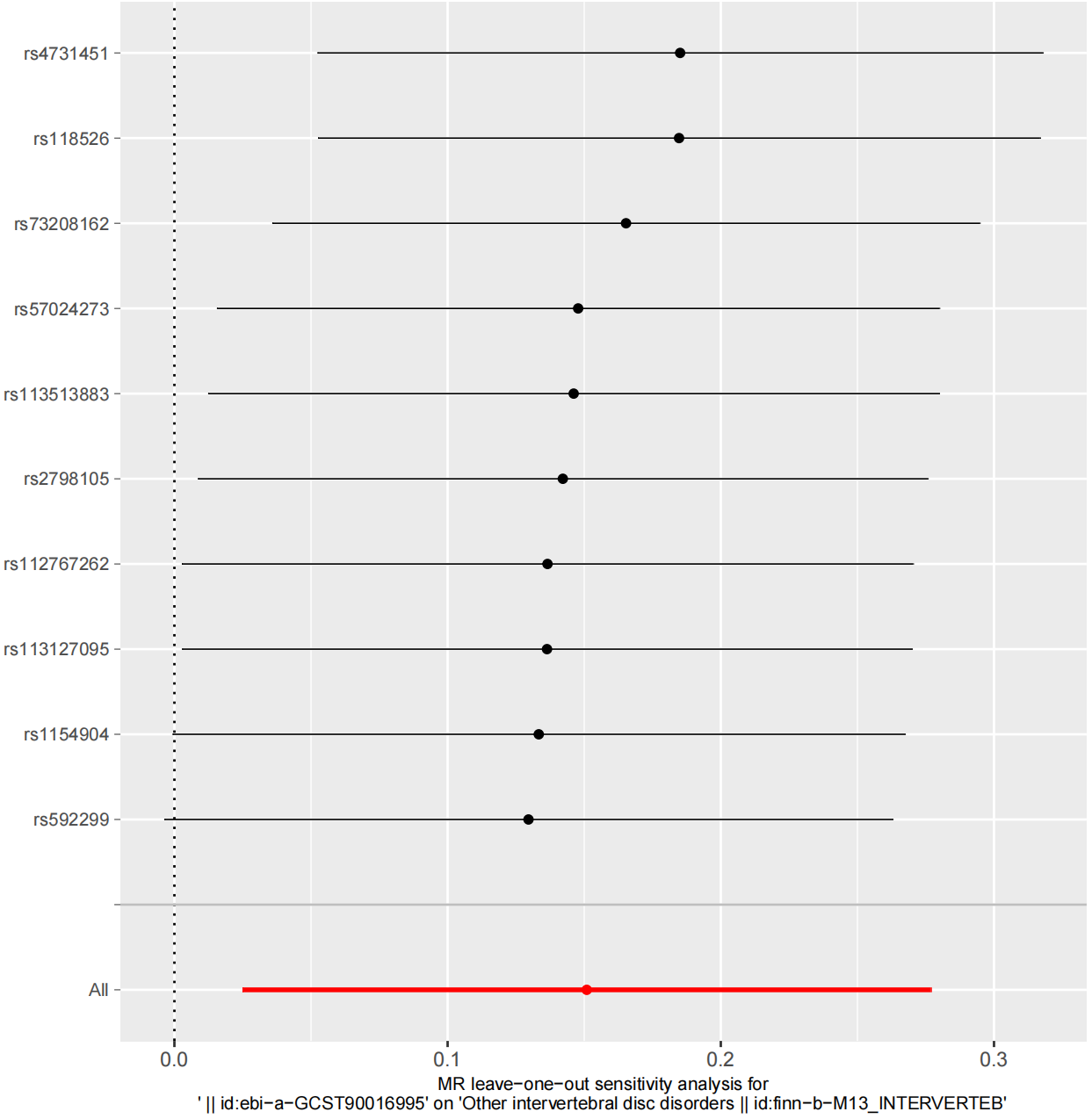

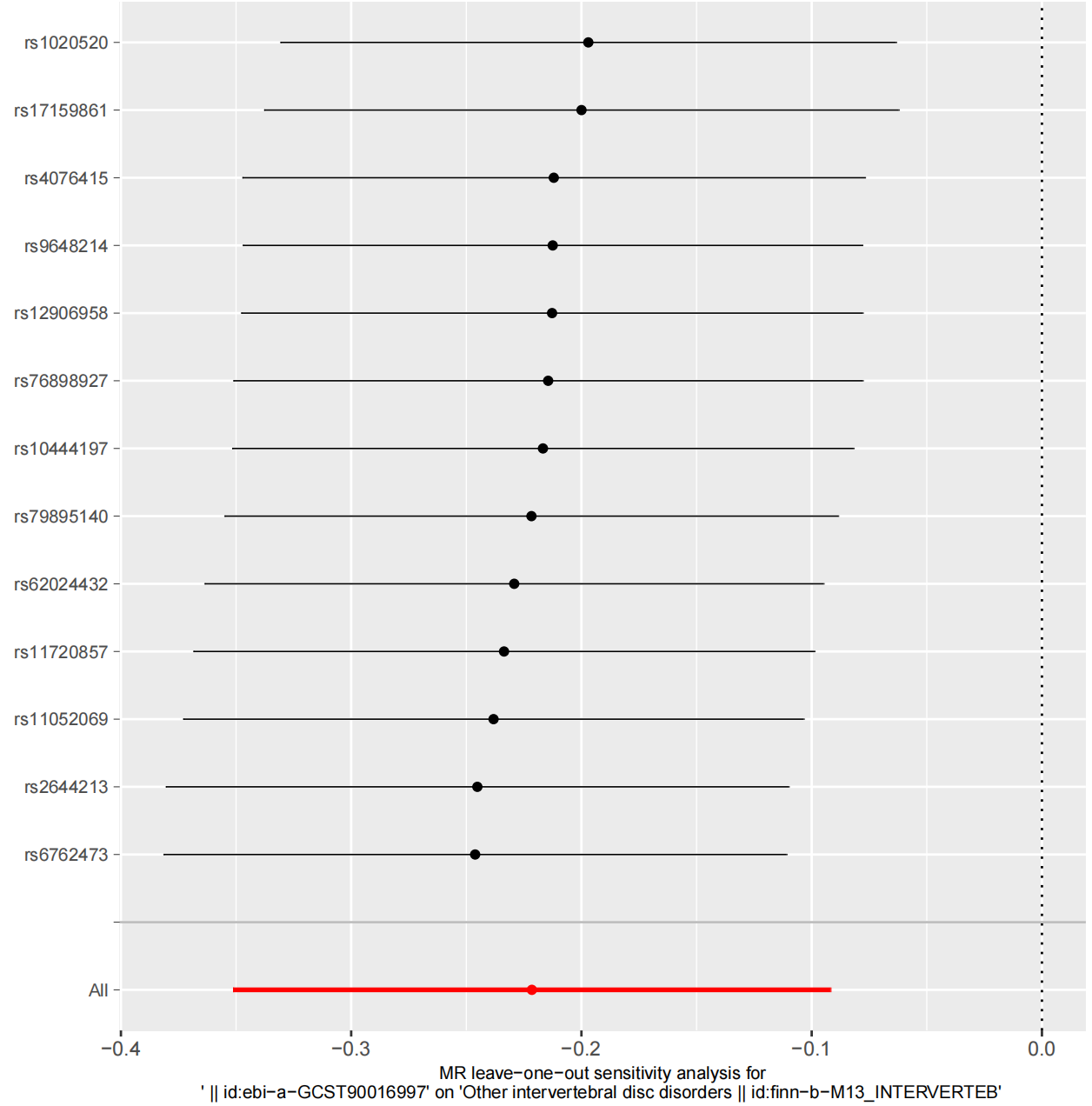

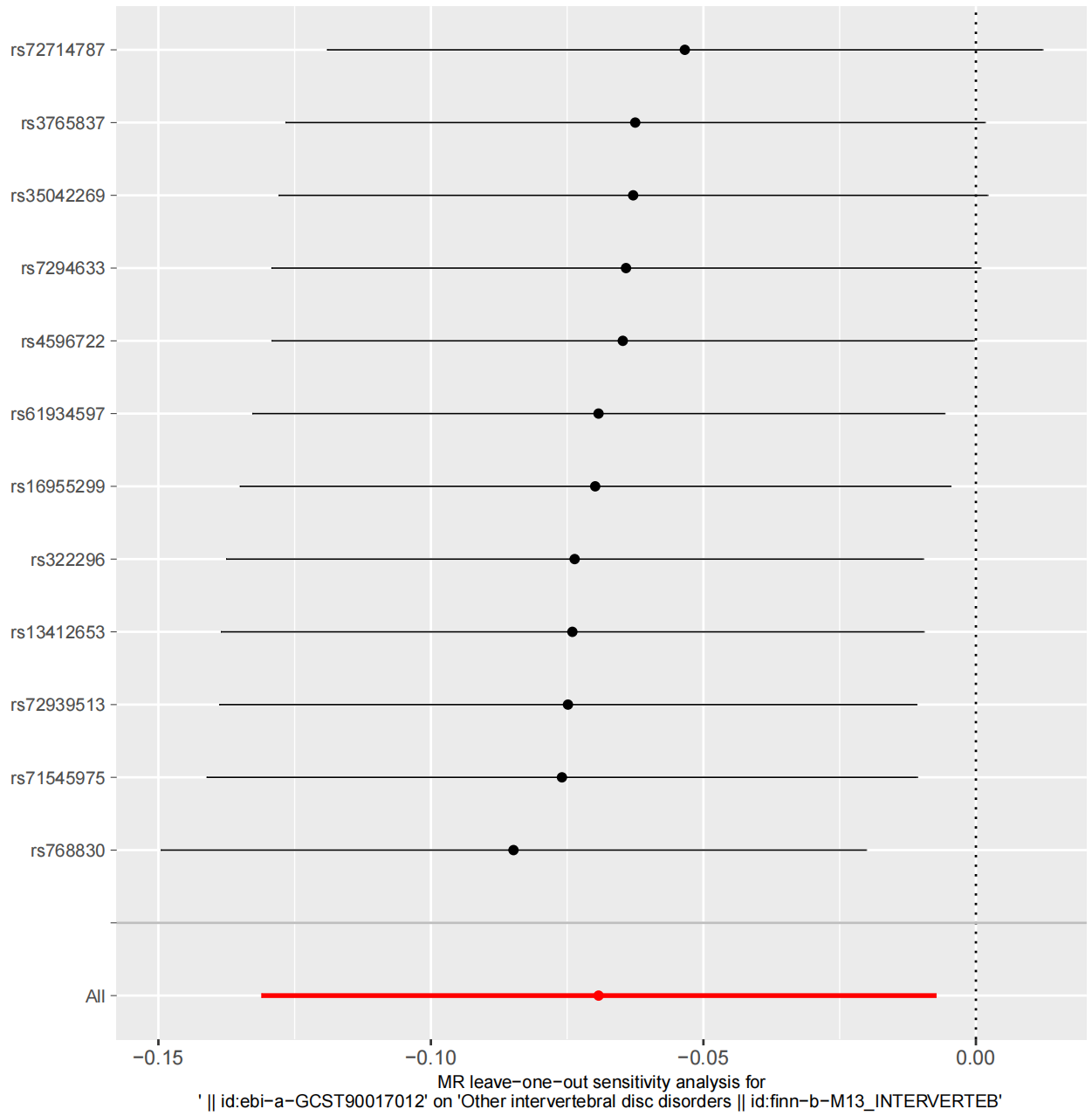

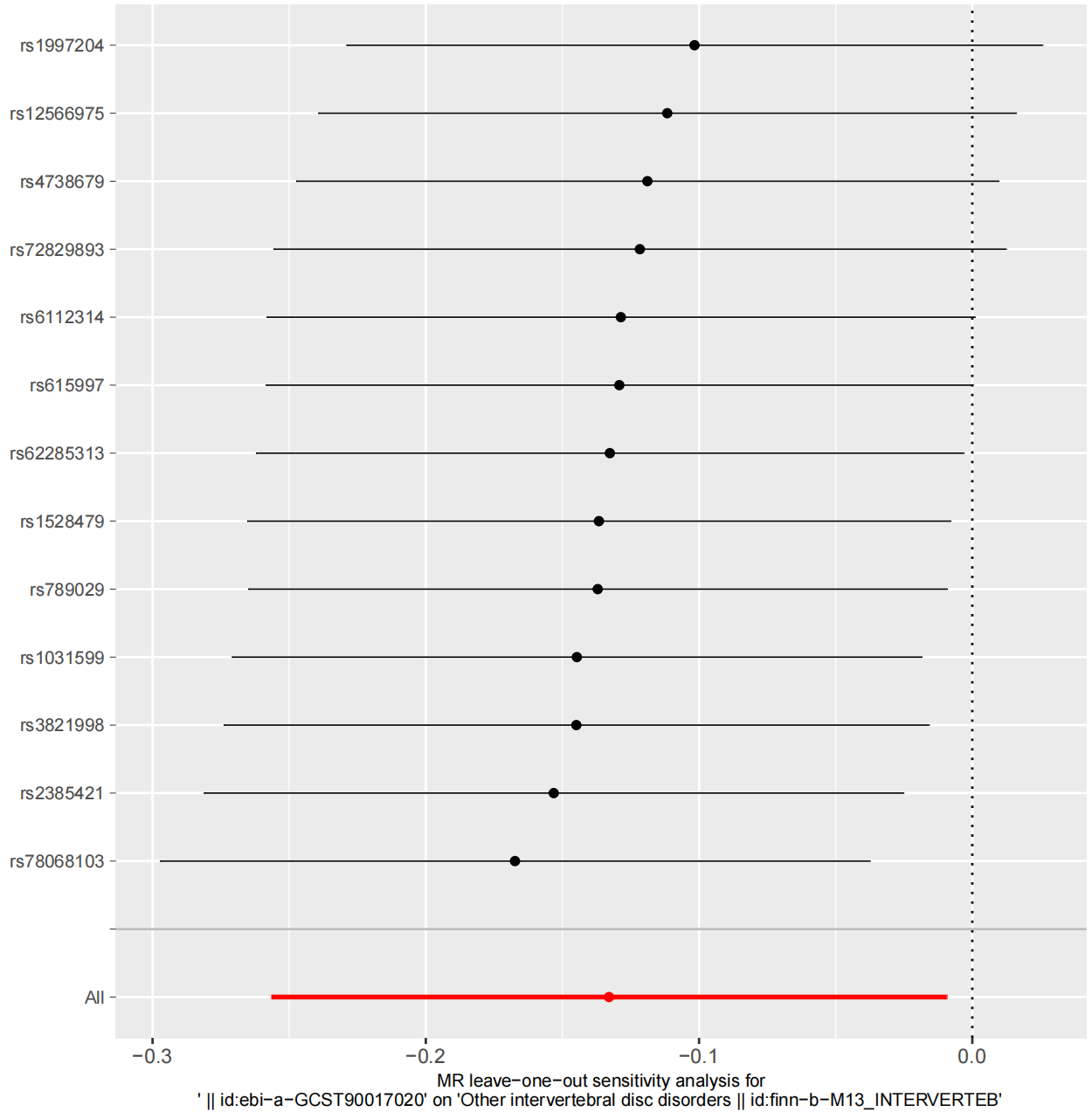

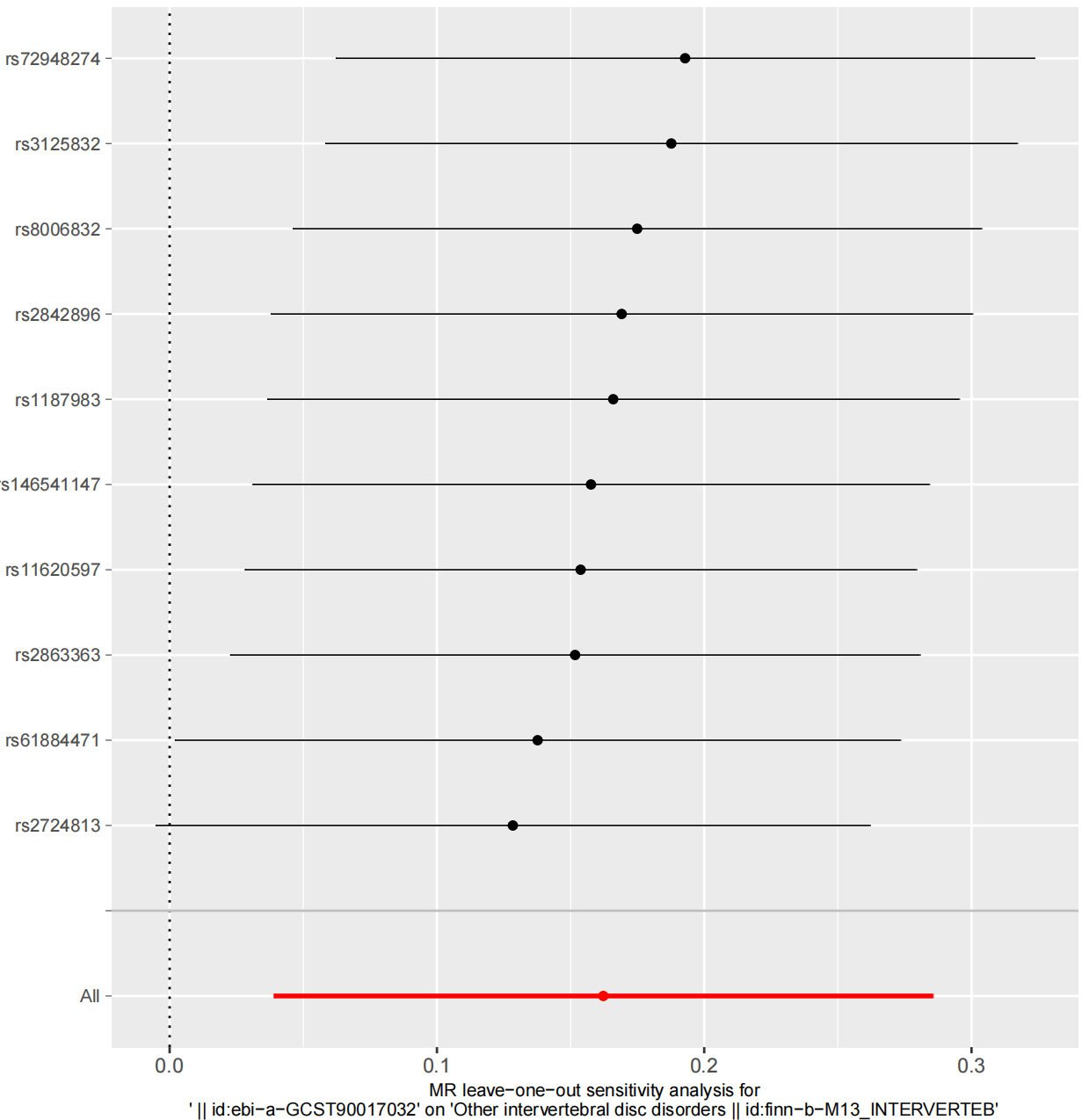

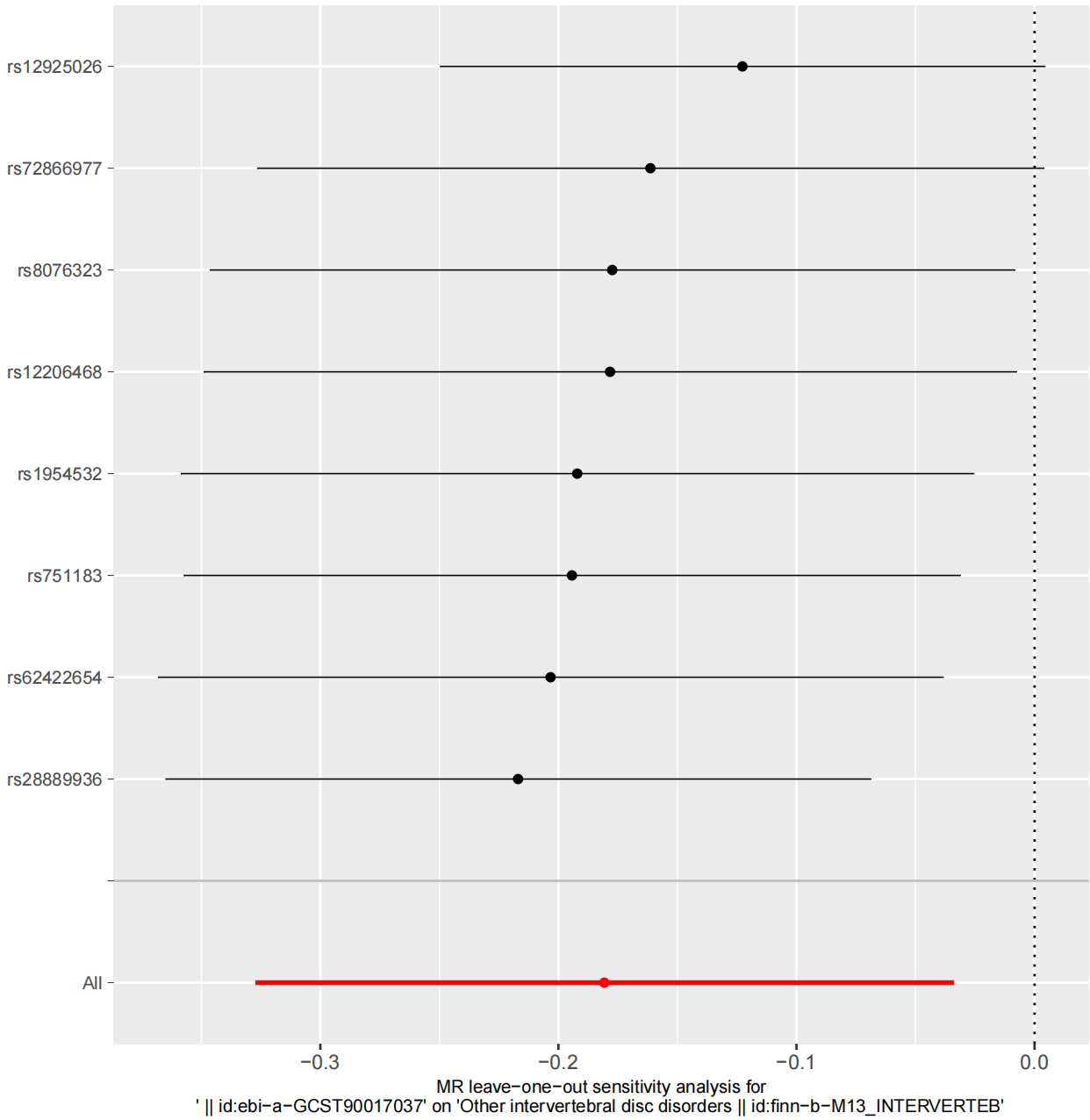

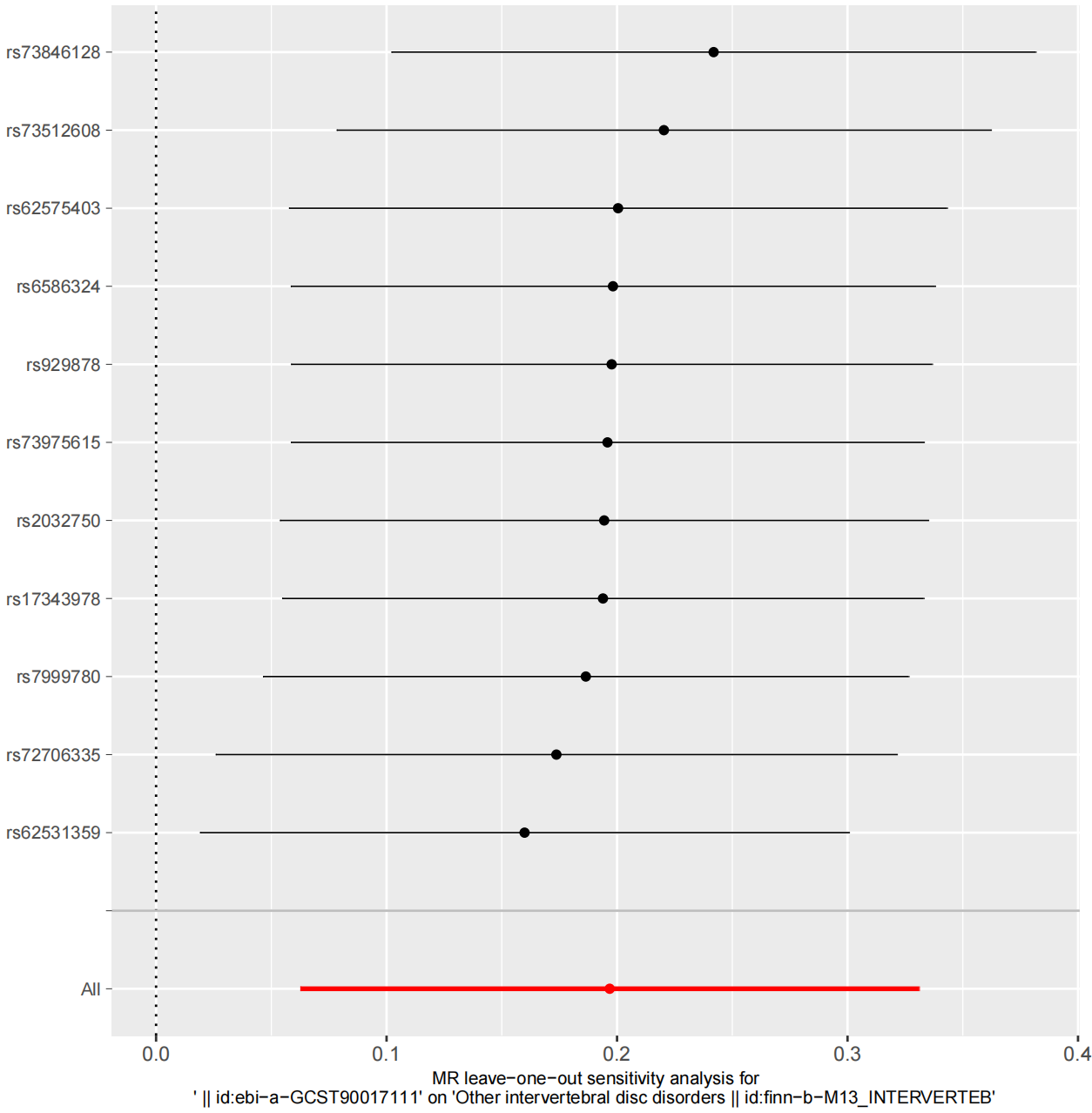


Supplementary figure1:Leave-one-out analysis for 9 GM taxa on IVDD

Supplement: Supplementary file 2 — Additional file 2. Fig. S1. Leave-one-out analysis for 9 GM taxa on IVDD. [file 13018_2023_4081_MOESM2_ESM.docx]
